# Intermediate Care Units in Europe and Italy: A Review of Structure, Outcomes, and Policy Implications for Internal Medicine

**DOI:** 10.3390/jcm14186543

**Published:** 2025-09-17

**Authors:** Gianni Turcato, Arian Zaboli, Alessandro Cipriano, Andrea Montagnani, Vieri Vannucchi, Filippo Pieralli, Anna Belfiore, Filippo Valbusa, Massimo Marchetti, Paolo Ferretto, Lucia Filippi, Antonio Voza, Lorenzo Ghiadoni, Walter Ageno, Christian J. Wiedermann

**Affiliations:** 1 Department of Internal Medicine, Intermediate Care Unit, Hospital Alto Vicentino (AULSS7), 36014 Santorso, Italy; massimo.marchetti@aulss7.veneto.it (M.M.); paolo.ferretto@aulss7.veneto.it (P.F.); lucia.filippi@aulss7.veneto.it (L.F.); 2 Health Professions Management, South Tyrolean Health Authority (SABES-ASDAA), 39100 Bolzano, Italy; zaboliarian@gmail.com; 3 Emergency Department, Nuovo Santa Chiara Hospital, Azienda Ospedaliero-Universitaria Pisana, 56100 Pisa, Italy; alessandrocipriano@gmail.com; 4 Department of Internal Medicine, Hospital Misericordia, 58100 Grosseto, Italy; montagnaniand@gmail.com; 5 Department of Internal Medicine, Santa Maria Nuova Hospital, 50122 Florence, Italy; vieri.vannucchi@gmail.com; 6 Intermediate Care Unit, Careggi University Hospital, 50134 Florence, Italy; filippopieralli@gmail.com; 7 Division of Internal Medicine, Clinica Medica “Augusto Murri”, Department of Biomedical Sciences and Human Oncology, University of Bari Medical School, 70124 Bari, Italy; belfiore.murri@gmail.com; 8 Division of Internal Medicine, Istituto di Ricovero e Cura a Carattere Scientifico Sacro Cuore-Don Calabria, 37024 Negrar, Italy; filippo.valbusa@sacrocuore.it; 9 Department of Biomedical Sciences, IRCCS Humanitas Research Hospital, 20072 Milano, Italy; antonio.voza@humanitas.it; 10 Department of Clinical and Experimental Medicine, University of Pisa, 56100 Pisa, Italy; lorenzo.ghiadoni@unipi.it; 11 Department of Internal Medicine, University of Padova, 35100 Padova, Italy; walter.ageno@unipd.it; 12 Institute of General Practice and Public Health, Province College for Health-Care Professions “Claudiana”, 39100 Bolzano, Italy; christian.wiedermann@am-mg.claudiana.bz.it

**Keywords:** intermediate care, IMCU, subintensive care, internal medicine, ICU utilization, hospital organization, healthcare policy

## Abstract

**Background/Objectives:** Intermediate Care Units (IMCUs) provide a level of care between general wards and Intensive Care Units (ICUs). While widely implemented across Europe, their use in the Italian internal medicine remains limited. To review the clinical effectiveness, organizational benefits, and policy relevance of IMCUs in Europe and assess opportunities and barriers to their implementation in the Italian hospital system. **Methods:** A narrative review of international and Italian literature from the origin of intermediate care models in 2025, with emphasis on patient outcomes, ICU utilization, cost-effectiveness, and governance models for IMCUs. **Results:** European studies consistently show that IMCUs improve patient flow, reduce ICU burden, and may reduce mortality among selected high-acuity patients. In Italy, respiratory and cardiac IMCUs have demonstrated similar benefits. However, general internal medicine IMCUs remain underdeveloped. The COVID-19 pandemic exposed structural gaps in the capacity for intermediate care. Recent legislative efforts (e.g., Decree-Law 34/2020) have aimed to expand sub-intensive care, but implementation is still heterogeneous. **Conclusions:** IMCUs are a cost-effective and clinically valuable strategy for managing non-ICU high-acuity patients. Structured integration of IMCUs into internal medicine in Italy could improve care quality and system efficiency. Clear triage protocols, adequate staffing, and strong organizational leadership are essential for success.

## 1. Introduction

Intermediate Care Units (IMCUs) offer a structured level of care positioned between the general ward and intensive care unit (ICU) [1]. These units are designed to manage patients who are too critically ill for standard ward care but do not require the comprehensive interventions provided by an ICU. IMCUs also serve as a transitional step for patients recovering from ICU care before they are transferred to a regular ward, thereby ensuring continuity of monitoring and treatment [1,2]. Typically, patients in an IMCU require support for single or multiple organ systems without the immediate need for invasive organ replacement therapies such as endotracheal intubation or renal replacement therapy. This includes individuals experiencing acute respiratory failure, sepsis, cardiac events, or other medical emergencies in which the clinical condition is unstable but potentially reversible with close observation and timely intervention [2].

The primary objective of IMCUs is to enhance resource allocation and clinical outcomes by providing a monitored environment with higher nurse-to-patient ratios and technical capabilities than general wards, yet with lower intensity and cost than that of ICUs. This approach facilitates earlier intervention, prevents unnecessary ICU admissions, and ensures smoother transitions between levels of care [1,2].

IMCUs have become an integral component of hospital critical care systems in several European nations. In 2007 an international survey indicated that 31% of hospitals across 75 countries had established an IMCU [3]. Countries such as the United Kingdom, Germany, Spain, and the Netherlands have developed formal policies and national guidelines concerning IMCU design, staffing, and function [4,5]. Spain has been notably active in IMCU research and dissemination [4].

**Figure 1:**
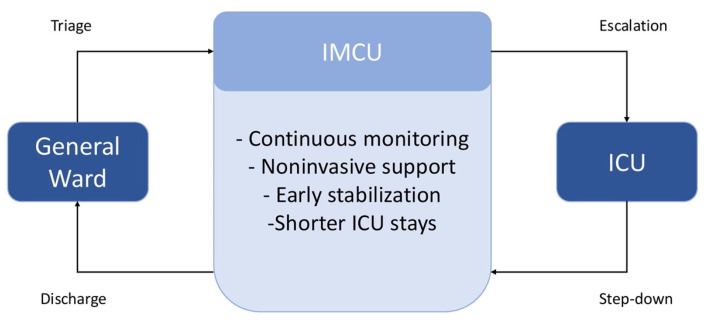
Role of Intermediate Medical Care Units (IMCUs) in the hospital care continuum. This schematic illustrates the functional position of IMCUs within the hospital care continuum, acting as a bridge between general wards and Intensive Care Units (ICUs).

Conversely, Italy has been slow to implement general medical IMCUs, with most intermediate care efforts historically confined to specialized settings, such as respiratory, cardiac or stroke units [6]. This narrative review synthesizes the available evidence on the clinical and organizational effectiveness of IMCUs and explores policy and implementation strategies pertinent to the Italian hospital system. Drawing on international experience, this review underscores the potential of IMCUs to enhance patient flow, alleviate ICU pressure, and provide high-quality care for acutely ill medical patients in internal medicine departments (Figure 1).

## 2. Methods

This manuscript is based on a structured narrative review of the literature, aiming to synthesize the current evidence on IMCUs in Internal Medicine, with a particular focus on their clinical effectiveness, organizational impact, and policy relevance in internal medicine.

### 2.1. Search Strategy and Scope

A comprehensive search of the biomedical literature was conducted across the PubMed/MEDLINE and EMBASE databases using combinations of the following keywords: “intermediate care unit,” “step-down unit,” “high-dependency unit,” “IMCU,” “subintensive care,” “internal medicine,” “governance,” “triage,” “cost-effectiveness,” and “Italy.” References from key review articles were screened for additional eligible sources. To provide historical and policy context, studies published between the inception of intermediate care models and 2025 were considered, including original research articles, systematic reviews, cohort studies, cost analyses, and national policy documents. Non-English publications were included if relevant.

### 2.2. Inclusion and Exclusion Criteria

Studies were included if they reported clinical outcomes (e.g., mortality, ICU transfers) in IMCU or step-down settings, health system effects (e.g., ICU utilization, staffing), patient- or staff-reported outcomes (e.g., satisfaction, well-being), and implementation strategies related to IMCUs within internal medicine units. Studies focusing on postoperative units were excluded unless their findings provided cross-sectorial insights (e.g., patient flow or ICU decongestion).

### 2.3. Data Synthesis and Structure

This review integrates findings to inform IMCU policy and planning in internal medicine contexts, without a formal meta-analysis due to heterogeneous study designs and outcomes. Evidence was summarized across five domains: clinical outcomes and mortality, stabilization and ICU resource utilization, patient and staff satisfaction, cost-effectiveness, and policy, governance, and implementation frameworks. To contextualize Italian developments, legislative documents (e.g., Decree-Law No. 34/2020) and national IMCU experiences were included in the European data. Reference consistency was verified using journal indexing and citation metadata.

## 3. Clinical Effectiveness of IMCUs and Patient Outcomes

### 3.1. Impact on Mortality and Morbidity

Recent evidence supports the clinical efficacy of IMCUs in improving outcomes for acutely ill medical patients. A comprehensive multicenter cohort study conducted across 17 countries, involving 5834 ICU patients, demonstrated that hospital mortality rates were significantly lower in facilities equipped with an IMCU, with a 37% reduction in the adjusted odds of death (OR 0.63) compared to hospitals lacking such units [6]. The survival advantage was particularly pronounced among patients requiring intensive therapy, indicating that IMCUs enhance ICU capacity by facilitating improved triage and earlier step-downs [1]. Similarly, a nationwide study utilizing a Japanese database revealed that patients admitted to IMCUs in hospitals without an ICU exhibited a higher risk of in-hospital mortality (adjusted OR 1.15; 95% CI: 1.10–1.20), underscoring the importance of co-location and integration with ICUs for effective care escalation [7].

In Italy, where general IMCUs are relatively rare, specialized intermediate units, particularly those focused on respiratory care, have shown positive outcomes. The establishment of respiratory IMCUs has resulted in reduced in-hospital mortality and decreased ICU admissions for patients experiencing acute respiratory failure, including those with Chronic Obstructive Pulmonary Disease (COPD) exacerbations and severe pneumonia [8]. Furthermore, research indicates that high-risk patients treated in step-down settings, as opposed to general wards, experience improved complication rates and enhanced pain management [9,10]. Notably, safety outcomes remain favorable since, even in complex cases, respiratory IMCUs maintain low mortality rates without an increase in adverse events [11].

A recent Italian quasi-experimental study found that the establishment of a general medical IMCU was associated with a 58% reduction in the relative risk of 30-day mortality in adjusted models (IRR 0.417; 95% CI: 0.388–0.449; *p* < 0.001), suggesting a protective effect through better patient allocation and early intervention [12].

### 3.2. Impact on Acute Stabilization

Timely stabilization of acute conditions is critical for improving the patient prognosis [13]. While this principle is well established in dedicated units such as cardiac and stroke care [14,15], emerging data suggest that general IMCUs may play a similar role in internal medicine departments. One Italian study showed that stabilization within 72 h was an independent protective factor for 30-day mortality (OR 0.115; 95% CI: 0.045–0.292; *p* < 0.001) and that early stabilization was more likely in IMCU patients than in those treated on wards (78.5% vs. 67.1%; *p* = 0.023). IMCU care was also an independent predictor of early stabilization (OR 2.283; 95% CI: 1.297–4.016; *p* = 0.004) [16].

### 3.3. Effect on ICU Utilization and Patient Flow

IMCUs play a crucial role in mitigating ICU congestion by managing patients who are on the cusp of requiring ICU admission, thereby optimizing the allocation of critical care resources [1,17]. Empirical evidence indicates that the introduction of an IMCU enables ICUs to admit patients with greater severity levels without a corresponding rise in ICU mortality rates, reflecting enhanced triage and capacity management [18].

Within the context of internal medicine, IMCUs have been shown to decrease the necessity for ICU transfers. The quoted quasi-experimental study conducted in Italy reported a 52.5% reduction in ICU transfers post-IMCU implementation (IRR, 0.475; 95% CI: 0.402–0.562; *p* < 0.001) [12]. Furthermore, IMCUs facilitate the discharge process from ICUs by offering monitored step-down care, which reduces the ICU length of stay and enhances patient flow from emergency departments and surgical theaters.

Historical data corroborate these findings. For instance, one study documented a decline in ward mortality following the establishment of an IMCU, suggesting that patients at risk of deterioration received more effective monitoring and treatment [19]. In smaller hospitals that lack ICU capacity, IMCUs may even obviate the need for patient transfers to other facilities while maintaining safety [20]. During the COVID-19 pandemic, IMCUs were instrumental in alleviating ICU demand, particularly for cases requiring respiratory support in overburdened systems, such as those in Spain [21,22,23].

### 3.4. Patient and Family Satisfaction, Comfort, and Quality of Life

Although clinical outcomes, such as mortality, remain the primary focus of most IMCU studies, there is a growing recognition of the importance of patient-centered outcomes as indicators of care quality [24]. Patients and their families often appreciate IMCUs for providing close clinical monitoring in a less restrictive and intimidating environment than ICUs, thereby enhancing comfort and satisfaction. One study reported improved patient and family satisfaction scores following the implementation of an IMCU, attributing this improvement to enhanced continuity of care and better communication, particularly when patients were no longer transferred between distant ICU and ward locations [24].

The IMCU environment is generally quieter and less over-stimulating than the ICU, which may positively affect mental well-being and reduce stress. Research indicates that optimizing environmental factors, such as minimizing alarm noise and nighttime disturbances, can significantly enhance patients’ perceived comfort in IMCUs [4].

Although direct evidence on long-term quality of life (QoL) remains limited, early initiation of noninvasive support and facilitated mobilization in IMCUs may help preserve functional status and mitigate complications such as deconditioning or delirium. Thus, intermediate care appears to support a more patient-friendly trajectory without compromising clinical outcomes.

Additionally, nursing staff in IMCUs often report higher job satisfaction than their colleagues in general wards or ICUs, likely due to improved working conditions, clinical autonomy, and continuity of care responsibilities [25]. A more satisfied and less stressed workforce may, in turn, contribute to better patient experience and outcomes. For context, Table 1 provides a concise side-by-side overview of care levels.

### 3.5. Safety and Limitations

The clinical and organizational advantages of IMCUs are contingent upon the appropriate selection of patients and timely escalation of care when necessary. A significant risk associated with IMCUs is the delayed transfer to the ICU; if a patient’s condition deteriorates while in the IMCU, any delay in escalation can lead to adverse outcomes. Research indicates that patients who require “rescue” transfer from the IMCU to the ICU exhibit higher mortality rates than those directly admitted to the ICU, often due to an initial underestimation of illness severity or delayed recognition of clinical deterioration [26]. This underlines the critical importance of implementing clear and consistent triage criteria. The IMCUs should not serve as a holding area for patients requiring immediate intensive care.

One significant concern, particularly highlighted in early discussions regarding the implementation of IMCUs, is the potential for fragmentation of care. When IMCUs function independently from ICU teams, the absence of coordination may compromise the continuity of care and elevate the risk of communication errors or delays during patient handovers [27]. To mitigate this issue, contemporary IMCU models in Europe prioritize organizational integration, frequently situating IMCUs under the same departmental governance as ICUs and establishing formal outreach structures. Examples of such structures include rapid response teams and joint clinical protocols that facilitate the timely assessment and transfer of deteriorating patients from general wards to IMCUs or ICUs.

In conclusion, IMCUs appear to provide a safe and effective care environment for high-acuity patients who do not require full ICU support, provided that robust triage systems, interdisciplinary coordination, and timely escalation mechanisms are established.

## 4. Cost-Effectiveness Considerations

A primary justification for IMCUs is their potential to deliver cost-effective care. Indeed, treatment within IMCUs is typically less costly than ICU care, while yielding superior outcomes compared to standard ward care. By maintaining lower nurse-to-patient ratios and utilizing less invasive monitoring and support, IMCUs can significantly decrease per-patient expenses [1]. For instance, managing a stable high-risk patient with telemetry and non-invasive ventilation (NIV) in an IMCU is considerably more economical than occupying a fully equipped ICU bed with one-to-one nursing and mechanical ventilation [28].

However, empirical evaluations of the cost-effectiveness of IMCUs have produced mixed outcomes. One concern is that IMCUs may inadvertently elevate total hospital costs by expanding the bed capacity and admitting patients who previously would not have received intensive monitoring. A study conducted in the Netherlands, for example, reported an increase in the average hospital costs per patient following IMCU implementation. This increase was attributed not to higher per-day costs but to a broader inclusion of high-acuity patients receiving more intensive care than they otherwise would have received [29].

Conversely, numerous studies have demonstrated the economic benefits associated with the use of IMCUs in preventing ICU admissions or complications. A recent cost analysis conducted in the Netherlands estimated annual savings of approximately €1.56 million attributable to the IMCU’s role in managing patients who would otherwise require more expensive ICU stays [30]. Importantly, these savings were realized only in healthcare systems with well-defined triage processes that ensured appropriate allocation between the ICU, IMCU, and general wards. Similarly, a study conducted in Spain reported “considerable economic savings” following the establishment of a respiratory IMCU, noting reductions in hospital stays and avoided ICU admissions while maintaining low mortality rates [11].

Other studies further support these results. A Canadian study indicated that many patients with myocardial infarction could be discharged directly from coronary IMCUs without increased morbidity, thereby shortening the length of stay and reducing overall treatment costs [31]. IMCUs also contribute to systemic cost savings by preventing ICU overcrowding and reducing the need for costly emergency transfers to other facilities when ICU beds are unavailable. They provide flexible surge capacity and help avert downstream complications, such as ventilator-associated pneumonia or ICU delirium by facilitating early noninvasive interventions.

Italy’s experience with respiratory intermediate care units (RIMCUs) substantiates the argument for their cost effectiveness. These units have demonstrated the capacity to decrease mortality rates and reduce ICU admissions for acute respiratory failure, thereby suggesting substantial cost savings by managing appropriate patients outside the ICU setting [32,33].

However, these cost savings are contingent on proper integration. If IMCU care merely adds an additional layer of hospitalization without replacing ICU time or preventing complications, the cumulative costs may increase without corresponding benefits [1]. Therefore, IMCUs are most cost-effective when implemented as part of a coordinated acute care model that ensures efficient patient allocation and avoids unnecessary duplication.

Table 2 provides a structured overview of the main domains in which IMCUs have demonstrated benefits, summarizing the evidence supporting clinical outcomes, system efficiency, and patient experience.

In conclusion, IMCUs can enhance the efficiency of health systems by aligning the intensity of care with patient needs and minimizing unnecessary utilization of ICUs. They also help mitigate the higher costs associated with inadequate monitoring of general wards. Evaluations in Europe largely endorse IMCUs as both cost-containment and quality-improvement strategies. Conversely, the limited adoption of IMCUs in Italian internal medicine may lead to inefficient resource utilization, either by overburdening ICUs with moderately ill patients or compromising outcomes in general wards. In Italy policymakers are currently exploring the implementation of IMCUs to reduce critical care expenditures while enhancing care delivery [34]. To realize this potential implementation, efforts must focus on reducing ICU demand and preventing avoidable complications through effective patient selection and integration of the technology.

Nonetheless, it should be emphasized that the economic advantages of IMCUs are not guaranteed. Their cost-effectiveness strongly depends on rigorous admission and discharge criteria, efficient patient flow, and the demonstrable substitution of more expensive ICU care. In the absence of these conditions, IMCUs may simply add an intermediate layer of care without achieving meaningful financial benefits.

## 5. Implementation

**Figure 2:**
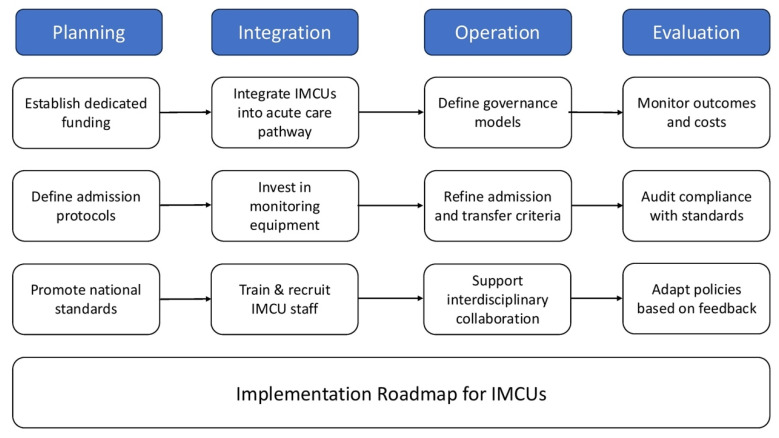
Implementation roadmap for IMCUs in Internal Medicine.

Figure 2 summarizes the key phases and operational domains involved in the successful implementation of IMCUs within internal medicine, based on the current European experience and emerging Italian frameworks.

### 5.1. Policy Directions

Recent European experiences increasingly advocate for the incorporation of Intermediate Medical Care Units (IMCUs) as a standard element within the hospital care continuum. Policy recommendations underscore the formal integration of IMCUs, often termed “Level 2” care beds, into hospital infrastructure and critical care planning. For instance, the modernization initiative of the UK’s National Health Service (NHS) in the early 2000s explicitly promoted the expansion of high-dependency care to avert the inappropriate treatment of critically ill patients in standard hospital wards [35]. In numerous healthcare systems, hospital beds are now categorized based on the required intensity of care rather than a simple ward-versus-ICU classification.

In 2018, the German Interdisciplinary Association for Intensive Care issued comprehensive guidelines on IMCU staffing and equipment, emphasizing that such units should be acknowledged as part of formally accredited critical care services [5,36]. Similarly, a European cohort study recommended that hospitals lacking IMCUs consider their establishment, given the observed clinical benefits of IMCUs [6].

Current evidence advocates for the expansion of IMCUs in both high-volume tertiary hospitals and smaller regional centers. While larger hospitals may benefit from dedicated units to manage fluctuating acuity levels, smaller institutions can utilize IMCUs to stabilize critically ill patients at an early stage, thereby guiding appropriate transfer or continued in-house care of these patients.

In Italy, efforts to integrate IMCUs into internal medicine are gaining momentum. This approach builds on positive experiences with specialized respiratory and cardiac units but extends further by emphasizing broad applicability across acute medical conditions. Unlike disease-specific intermediate units, internal medicine IMCUs are optimally positioned to serve as hubs for early stabilization, thereby improving patient flow, facilitating triage, and enhancing resource utilization.

Functional and organizational integration of Emergency Departments, IMCUs, and ICUs is essential. The establishment of shared clinical pathways facilitates more coordinated care for patients with high acuity who do not require full intensive support. Furthermore, this integration enhances system-wide outcomes by streamlining transitions between care levels and minimizing avoidable delays.

Policy frameworks should also mandate the establishment of standardized criteria for IMCU admission and discharge. An additional challenge is the absence of universally agreed standards for IMCUs. Nurse-to-patient ratios, required equipment, and even the spectrum of patients admitted vary considerably not only across countries but also between hospitals within the same system. This heterogeneity explains part of the inconsistency in reported outcomes and makes it difficult to define a single benchmark for clinical or economic performance. Strengthening international consensus on minimal structural and organizational requirements could therefore represent an important step toward more consistent implementation and evaluation. This includes the implementation of early warning scores, deployment of rapid response teams, and development of structured protocols for safe transfer between the emergency department, IMCU, and ICU. In the absence of clear triage processes, the potential clinical and economic, may be compromised.

### 5.2. Operational Requirements and Governance

#### 5.2.1. Investment in Equipment and Staff

Despite robust clinical and policy endorsements, the implementation of IMCUs encounters several practical challenges, with resource allocation being paramount. The establishment of a new unit necessitates capital investment in monitoring equipment, physical infrastructure, and, most critically, in human resources. Staffing IMCUs presents a particular challenge, as these units typically require a nurse-to-patient ratio of approximately 1:3, which is intermediate between ward care and ICU staffing levels [37]. Achieving this standard may require either the recruitment of additional personnel or the redistribution of existing staff, both of which are challenging in health systems that are already experiencing nursing shortages.

Beyond staffing ratios, the expertise of the IMCU personnel is crucial. Nurses and physicians must be trained to identify early clinical deterioration and manage advanced interventions, such as noninvasive ventilation (NIV), high-flow oxygen therapy, and continuous intravenous drug infusions [38]. In countries such as Italy, where ICU staffing levels are already strained, securing qualified staff for a dedicated IMCU, particularly under a closed-provider model, can be a significant obstacle [25].

#### 5.2.2. Governance Models and Structural Integration

Another fundamental issue in implementation pertains to governance: determining which department should oversee a medical IMCU. Governance models exhibit considerable variation across Europe. Some IMCUs are managed by intensivists, whereas others are overseen by internists or subspecialists, such as pulmonologists, particularly in units with a respiratory focus [39,40].

A recent systematic review conducted in Europe confirmed this heterogeneity, indicating the absence of a singular dominant model concerning the structure, clinical leadership, or admission criteria [41]. This variability highlights the need for governance models that can be adapted to local contexts. Each governance structure offers distinct advantages: intensivist-led IMCUs provide a high level of critical care expertise, whereas internist-led units often enable earlier clinical engagement and improved integration with general ward pathways [12,20,42].

In Italy, positioning the IMCU under the leadership of internal medicine may offer significant advantages to the healthcare system. Given the aging hospital population in the country, characterized by high rates of multimorbidity and functional decline, clinical trajectories often prioritize stabilization and coordinated discharge planning over aggressive escalation. Therefore, integrating IMCUs within medical departments may more effectively address systemic challenges, such as acute care bed shortages, prolonged hospital stays, and inefficient discharge pathways. However, the successful dissemination of this model, both in hub hospitals and spoke facilities, requires dedicated training pathways for internal medicine specialists and nursing staff. Without structured educational programs, the model risks remaining a theoretical framework rather than becoming widespread clinical practice.

Conversely, governance models based solely within the Emergency Department (ED) may facilitate a more rapid response to acute presentations but risk compromising continuity of care throughout the inpatient stay period. Furthermore, staffing shortages and high volumes of low-priority presentations already overwhelms many Italian EDs. These constraints make it challenging to provide consistent structured stabilization for critically ill patients [16].

To mitigate these risks, the effective governance of IMCUs necessitates the establishment of clearly defined interdisciplinary protocols, transparent responsibilities, and shared accountability structures. Encouraging cross-departmental collaboration, as opposed to rigidly assigning IMCUs to a single service, can alleviate managerial conflicts and facilitate integration into the hospital’s broader acute care system [43].

The benefits of IMCUs are not inherent to their designation but are driven by a combination of structural and organizational factors. Core elements include an adequate nurse-to-patient ratio, the availability of advanced monitoring and noninvasive support tools, well-defined triage and escalation protocols, and staff expertise in the management of high-acuity patients. Without these conditions, the simple re-labeling of a ward as an “IMCU” is unlikely to yield the improvements in outcomes and system efficiency reported in the literature.

Ultimately, the optimal governance model should be tailored to each hospital’s size, case mix, and resource availability. Whether directed by emergency medicine, intensive care, internal medicine, or through joint governance, the IMCU must operate according to standardized clinical goals, triage criteria, and transfer protocols to ensure consistency and quality.

### 5.3. Admission Criteria and Key Factors for Feasibility

The effective implementation of IMCUs necessitates meticulous planning and adaptation to specific contexts. Insights from established European systems offer valuable guidance for integrating these units into the Italian healthcare framework. The primary challenge is establishing suitable admission criteria. If the thresholds are excessively lenient, IMCUs may become inundated with patients who could be adequately managed in general wards, thereby undermining their intended purpose and leading to inefficient use of resources. Conversely, if the criteria are overly stringent, clinically unstable patients may remain in general wards until their condition deteriorates to the point of requiring emergency transfer to the ICU, a situation correlated with poorer outcomes.

To ensure appropriate utilization, hospitals must implement structured triage protocols and early warning systems that facilitate the timely identification of patients who may benefit from IMCUs. These tools should be complemented by clear and standardized criteria for escalation to the ICU, enabling safe and efficient transitions across levels of care [44]. Importantly, IMCUs should be recognized as integral components of the acute care pathway rather than optional or auxiliary monitored areas. Their role in clinical stabilization, resource optimization, and system-wide patient flow warrants their inclusion in hospital infrastructure plans, with corresponding funding mechanisms and alignment with national accreditation and quality standards [5]. However, particular attention must be paid to the step-down process toward internal medicine and geriatric wards, which, if not carefully regulated, may risk overwhelming IMCU themselves, especially in hospitals lacking adequate post-IMCU capacity.

## 6. The Establishment of Sub-Intensive Care Units in Italy

The establishment of the “Unità di Terapia Subintensiva” marks a significant advancement in Italy’s efforts to enhance care for patients who require close monitoring and advanced support but do not meet the full criteria for ICU admission. Legislative measures, particularly Decree-Law No. 34/2020, provide a structural framework for the nationwide expansion of sub-intensive care capacity. Nevertheless, the implementation of these measures remains inconsistent, with considerable regional disparities in their adoption and resource allocation.

Historically, Italian hospitals have been slow to establish general medical IMCUs outside specialized settings. Acutely ill internal medicine patients have traditionally been managed either in general wards or transferred directly to ICUs, with limited structured step-down options available. This persistent gap in intermediate care became particularly evident during the COVID-19 pandemic, when the shortage of monitored beds contributed to ICU overload. Many moderately severe cases lack appropriate settings for noninvasive support, resulting in either premature ICU admissions or suboptimal management in general wards [34].

In response to this situation, the Italian government enacted Decree-Law No. 34 on 19 May 2020, commonly referred to as the “Decreto Rilancio” (Relaunch Decree), to strengthen the national health system by expanding the IMCU capacity, including the establishment of Sub-intensive Care Units (SIC). These units were specifically designed to serve as an intermediary level between general wards and ICUs, providing intensive monitoring and organ support outside the context of full critical care [45].

Under this decree, the government mandated the creation of 4225 sub-intensive care beds, equating to approximately seven beds per 100,000 inhabitants. This reform aimed to enhance interdisciplinary integration across internal medicine, pulmonology, cardiology, and anesthesiology, thereby promoting a more cohesive model of sub-intensive care [45].

Despite the policy commitment, implementation remains in its nascent and inconsistent stages. Numerous hospitals continue to encounter structural, organizational, and staffing challenges that impede the effective establishment of these units. Regional disparities in planning capacity, infrastructure, and workforce availability further complicate nationwide rollout [46]. Moving forward, the sustainability and clinical impact of sub-intensive care in Italy will depend on standardized policy frameworks, targeted investments, and robust integration with hospital-wide care pathways.

## 7. Limitations

This review has several limitations. First, although we followed a structured search strategy, the narrative review design inherently carries a risk of selection bias, as the inclusion of studies is not subject to the same systematic methodology and statistical synthesis of a formal meta-analysis.

Another limitation is the absence of internationally standardized definitions and operational criteria for IMCUs. Staffing ratios, equipment requirements, and cost structures vary widely across countries and even across hospitals within the same healthcare system. This heterogeneity complicates direct comparisons and makes it difficult to provide universally applicable benchmarks or cost estimates. As a result, findings from different contexts may not be directly transferable, and our review should be interpreted with this variability in mind.

## 8. Conclusions

**Figure 3:**
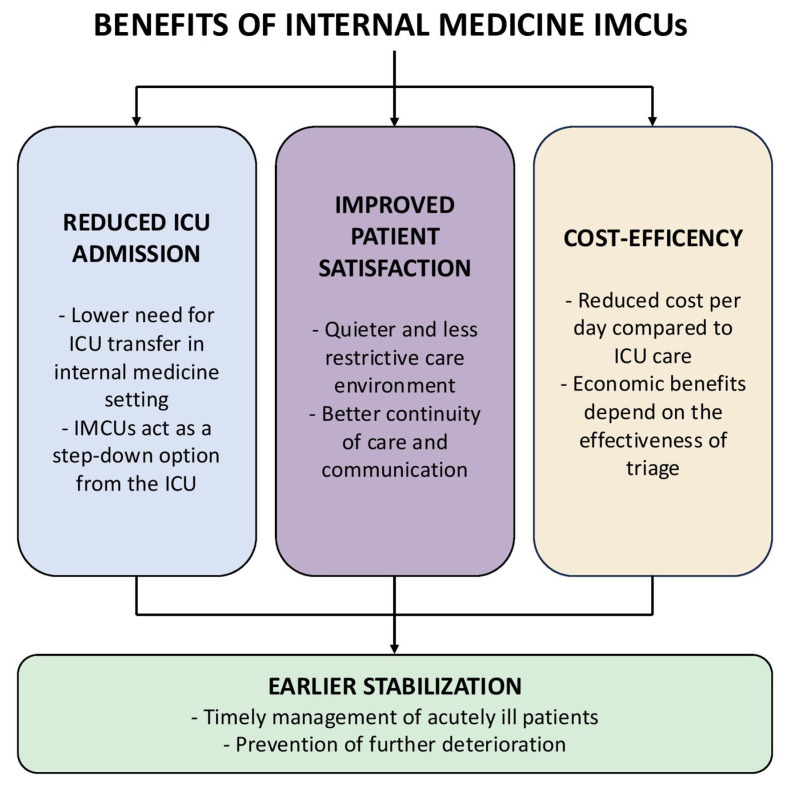
Benefits of IMCUs in Internal Medicine.

IMCUs integrated within medical departments have demonstrated significant clinical and organizational advantages across European healthcare systems and represent a promising strategy for enhancing acute care delivery in Italy (Figure 3). As a structural innovation, IMCUs provide a novel model for managing high-acuity patients who do not require full ICU support. This approach improves system-wide triage, alleviates ICU congestion, and enables general wards to focus on less complex care [1,6,23].

When effectively integrated into care pathways that connect Emergency Departments, ICUs, and hospital wards, IMCUs contribute not only to reduced mortality in selected patient populations [6] but also to enhanced patient and family satisfaction [4], improved staff well-being [47], and, under appropriate governance, potential cost savings [11,30]. Their ability to stabilize patients early in the course of acute illness is particularly valuable for time-sensitive conditions, with direct implications for outcomes and hospital efficiency [16]. However, several challenges remain. In the absence of clear triage criteria and organizational integration, IMCUs may inadvertently disrupt care continuity [48] or delay necessary ICU transfers, thereby increasing the clinical risk [26]. Successful implementation also relies on adequate staffing, targeted training, and consistent adherence to shared clinical protocols, factors that continue to present barriers in many institutions [49,50]. Moreover, the cost-effectiveness of IMCUs requires further investigation, particularly in settings where structural integration is weak and care transitions are fragmented.

Despite these concerns, the European experience illustrates that IMCUs, when meticulously designed and supported by appropriate investment, can substantially enhance patient safety, clinical outcomes, and operational efficiency. Countries such as Germany, Spain, and the United Kingdom have successfully integrated IMCUs as fundamental components of hospital infrastructure, yielding measurable improvements in patient flow and in system performance. In Italy, the delayed implementation of general medical IMCUs, compounded by the COVID-19 crisis, presents both challenges and opportunities. Establishing an effective IMCU network will necessitate not only financial resources and political commitment but also cultural transformation and strong institutional leadership. Nevertheless, the evidence strongly indicates that the advantages of IMCUs outweigh their limitations. Recent systematic reviews have confirmed that general medical IMCUs are increasingly essential for managing high-acuity non-ICU patients [41]. Their structural variability underscores the importance of flexible, activity-based admission criteria rather than rigid disease- or severity-based models [41]. This perspective has been echoed by leading European internists, who advocate for stronger involvement of internal medicine in IMCU development, standardized training, and integration into acute care hubs [51].

The integration of IMCUs into the contemporary acute care framework enhances resource allocation, decreases preventable mortality, and improves the perceived quality of care. Their development should be regarded not as a luxury but as a structural necessity, particularly during this period of healthcare system reform in the post-pandemic era. Transitioning beyond the binary ward–ICU model is no longer optional but essential.

## Figures and Tables

**Table 1:** Comparative overview of administrative, economic, and clinical features across levels of care (general medical ward, IMCU, and ICU).

Domain	General Medical Ward	IMCU	ICU
Positioning & scope	Standard ward care with basic monitoring for low–moderate acuity patients.	“Level-2” care between ward and ICU; manages high-acuity patients not requiring full invasive organ support.	“Level-3” critical care with advanced invasive organ support and continuous monitoring.
Governance/integration	Ward-led pathways; escalation to IMCU/ICU when needed.	Should be structurally integrated with ED and ICU, with shared triage/escalation criteria and pathways.	ICU under accredited critical-care governance; benefits when an IMCU exists for step-down.
Core technologies/therapies	Selective telemetry; standard oxygen therapy.	Continuous monitoring; NIV/HFNC; telemetry; protocolized triage/escalation.	Invasive mechanical ventilation, RRT, multi-organ support.
Admission criteria (typical)	Clinically stable; low risk of rapid deterioration.	High-acuity patients without immediate indication for invasive supports; standardized admission/discharge criteria recommended.	Organ failure requiring invasive support or immediate critical care.
In-hospital LOS	May be longer for high-acuity patients when enhanced monitoring/support are unavailable.	Can reduce hospital LOS through earlier stabilization and smoother pathways (shown in respiratory IMCUs).	LOS may increase if step-down capacity is lacking; improved when IMCU enables earlier transfer.
Primary clinical goal	Routine care and recovery/discharge.	Early stabilization to avoid escalation and to enable safe step-down from ICU.	Stabilize the most critically ill with advanced supports.

**Table 2:** Summary of Evidence for Clinical, System, and Patient-Level Benefits of Intermediate Care Units in Internal Medicine.

Domain	Clinical Benefit	System Efficiency	Patient Experience
Mortality reduction	Yes [6,12,16]	—	—
ICU decongestion	—	Yes [1,17,18,30]	—
Early stabilization	Yes [16]	Yes [13,16]	—
Satisfaction and well-being	—	—	Yes [24,25]
Cost-effectiveness	—	Mixed [29,30]	—

## Data Availability

No new data were generated.
